# Differential Contribution of Ca^2+^-Dependent Mechanisms to Hyperexcitability in Layer V Neurons of the Medial Entorhinal Cortex

**DOI:** 10.3389/fncel.2017.00182

**Published:** 2017-06-30

**Authors:** Eric C. Lin, Crescent L. Combe, Sonia Gasparini

**Affiliations:** ^1^Neuroscience Center of Excellence, Louisiana State University Health Sciences CenterNew Orleans, LA, United States; ^2^Department of Cell Biology and Anatomy, Louisiana State University Health Sciences CenterNew Orleans, LA, United States

**Keywords:** entorhinal cortex, dendrites, TRP channels, excitatory transmission, patch clamp electrophysiology, epileptogenesis, calcium channels, NMDA receptors

## Abstract

Temporal lobe epilepsy is characterized by recurrent seizures in one or both temporal lobes of the brain; some *in vitro* models show that epileptiform discharges initiate in entorhinal layer V neurons and then spread into other areas of the temporal lobe. We previously found that, in the presence of GABA_A_ receptor antagonists, stimulation of afferent fibers, terminating both at proximal and distal dendritic locations, initiated hyperexcitable bursts in layer V medial entorhinal neurons. We investigated the differential contribution of Ca^2+^-dependent mechanisms to the plateaus underlying these bursts at proximal and distal synapses. We found that the NMDA glutamatergic antagonist D,L-2-amino-5-phosphonovaleric acid (APV; 50 μM) reduced both the area and duration of the bursts at both proximal and distal synapses by about half. The L-type Ca^2+^ channel blocker nimodipine (10 μM) and the R- and T-type Ca^2+^ channel blocker NiCl_2_ (200 μM) decreased the area of the bursts to a lesser extent; none of these effects appeared to be location-dependent. Remarkably, the perfusion of flufenamic acid (FFA; 100 μM), to block Ca^2+^-activated non-selective cation currents (*I*_CAN_) mediated by transient receptor potential (TRP) channels, had a location-dependent effect, by abolishing burst firing and switching the suprathreshold response to a single action potential (AP) for proximal stimulation, but only minimally affecting the bursts evoked by distal stimulation. A similar outcome was found when FFA was pressure-applied locally around the proximal dendrite of the recorded neurons and in the presence of a selective blocker of melastatin TRP (TRPM) channels, 9-phenanthrol (100 μM), whereas a selective blocker of canonical TRP (TRPC) channels, SKF 96365, did not affect the bursts. These results indicate that different mechanisms might contribute to the initiation of hyperexcitability in layer V neurons at proximal and distal synapses and could shed light on the initiation of epileptiform activity in the entorhinal cortex.

## Introduction

Temporal lobe epilepsy is characterized by seizures in limbic structures, such as the hippocampus, amygdala and parahippocampal cortices. Early work on epileptogenic mechanisms focused on the hippocampus, but subsequent studies have identified fundamental roles of parahippocampal areas, including the entorhinal cortex (Scharfman, 2002). It has been proposed that a decrease in the inhibitory control exerted by the perirhinal and entorhinal cortices on the communication between neocortex and hippocampus could lead to hyperexcitability and ultimately to epilepsy in the limbic system (de Curtis and Paré, 2004).

Different pharmacological treatments, including GABA_A_ receptor antagonists, K^+^ channel blockers, low [Mg^2+^]_o_ and high [K^+^]_o_ have been used to mimic epileptiform synchronization in *in vitro* preparations (Jefferys, 1998; Avoli et al., 2002), suggesting that multiple mechanisms, both synaptic and intrinsic, may be implicated in epileptogenesis. Simultaneous extracellular recordings to analyze the onset of ictal discharges have shown that hypersynchronous activity initiates in the entorhinal cortex and then spreads to the hippocampus in low [Mg^2+^]_o_ or in the presence of bicuculline (Walther et al., 1986; Stanton et al., 1987; Jones and Lambert, 1990a; Wilson et al., 1998). Paired intracellular recordings have shown that interictal-like discharges are observed first within the network of layer V neurons and propagate to the superficial layers via axon collaterals (Jones and Lambert, 1990b). As such, EC layer V neurons could have a fundamental role in the development of epileptic activity in the temporal lobe.

Layer V principal cells are an heterogeneous population of neurons from a morphological and physiological point of view (Hamam et al., 2000). Interestingly, none of the neuronal subtypes display the intrinsic bursting behavior that has been proposed to act as a “pacemaker” for the initiation of epileptogenic events in other brain areas (Gutnick et al., 1982; Traub and Wong, 1982). The tendency of EC layer V neurons to fire in bursts under hyperexcitable conditions may instead reside in complex interactions among synaptic inputs and dendritic voltage-dependent conductances. Entorhinal layer V neurons receive a fairly well characterized excitatory input from the hippocampus at their proximal and basal dendritic locations (Swanson and Cowan, 1977; Sørensen and Shipley, 1979; Jones, 1987; Jones and Heinemann, 1988). On the other hand, only recently it has become clear that EC layer V neurons receive excitatory afferents to their distal dendritic regions (Medinilla et al., 2013), some of which originate in the pre- and parasubiculum and the postrhinal cortex (Canto et al., 2012; Koganezawa et al., 2015). Interestingly, we have previously found that the distal synapses generate hyperexcitable bursts more frequently than proximal synapses when inhibition is abolished (Medinilla et al., 2013). In the current work, we have therefore investigated whether differential mechanisms might be responsible for burst generation at proximal and distal synapses. We found that both synaptic (NMDA glutamatergic receptors) and intrinsic (voltage-dependent Ca^2+^ channels) mechanisms contribute to the plateaus underlying the hyperexcitable bursts. The most striking finding, however, was that the blockade of transient receptor potential (TRP) channel-mediated Ca^2+^-activated non-selective cation currents (*I*_CAN_) by flufenamic acid (FFA) abolished burst firing and switched the suprathreshold response to single action potentials (APs), only at proximal synapses. This effect was partially mimicked by a selective blocker for a subtype of TRP channels, 9-phenanthrol. Taken together, these results show that TRP channels are the major mechanisms responsible for burst generation at proximal, but not distal, dendritic locations in entorhinal layer V neurons, and could help identify new potential therapeutic targets in epilepsy.

## Materials and Methods

### Slice Preparation and Maintenance

Four hundred micrometer-thick slices containing the entorhinal cortex (EC) and the hippocampus were prepared from 7 week-old to 10 week-old male Sprague Dawley rats as previously described (Gasparini, 2011). This study was carried out in accordance with the recommendations of ethical principles established by the 1996 National Research Council in the “Guide for the Care and Use of Laboratory Animals”. The protocol was approved by the Institutional Animal Care and Use Committee at Louisiana State University Health Sciences Center in New Orleans. Rats were deeply anesthetized with an intraperitoneal injection of ketamine and xylazine (90 and 10 mg/Kg, respectively; additional doses were administrated until the toe-pinch reflex subsided completely), perfused through the ascending aorta with an ice-cold, oxygenated solution and decapitated. After removing the brain, horizontal slices were obtained using a vibratome; the slices were then placed in a holding chamber with oxygenated ACSF. After an incubation of at least 2 h, individual slices were transferred to a submerged recording chamber, where EC layer V principal cells were visualized using a Zeiss Axioskop fit with differential interference contrast (DIC) optics under infrared illumination. Only cells that showed a prominent apical dendrite emerging from the soma were selected for the recordings.

### Electrophysiological Recordings, Solutions and Chemicals

The external solution used for the recordings contained (in mM): NaCl, 125; KCl, 2.5; NaHCO_3_, 25; NaH_2_PO_4_, 1.25; CaCl_2_, 2; MgCl_2_, 1; dextrose, 25, and was saturated with 95% O_2_ and 5% CO_2_ at 34–36°C (pH 7.4). Whole-cell patch-clamp somatic recordings were performed in current-clamp configuration using a Dagan BVC-700 amplifier in the active “bridge” mode. Patch electrodes had a resistance of 2–4 MΩ and were filled with solution containing (in mM): K-methylsulphonate, 125; HEPES, 10; NaCl, 4; Mg_2_ATP, 4; Tris_2_GTP, 0.3; phosphocreatine, 14, (buffered with Tris-base to a pH of 7.3). Series resistance was monitored constantly using a hyperpolarizing current step, and was generally in the order of 7–20 MΩ; recordings were discarded when series resistance exceeded 30 MΩ.

**Figure 1:**
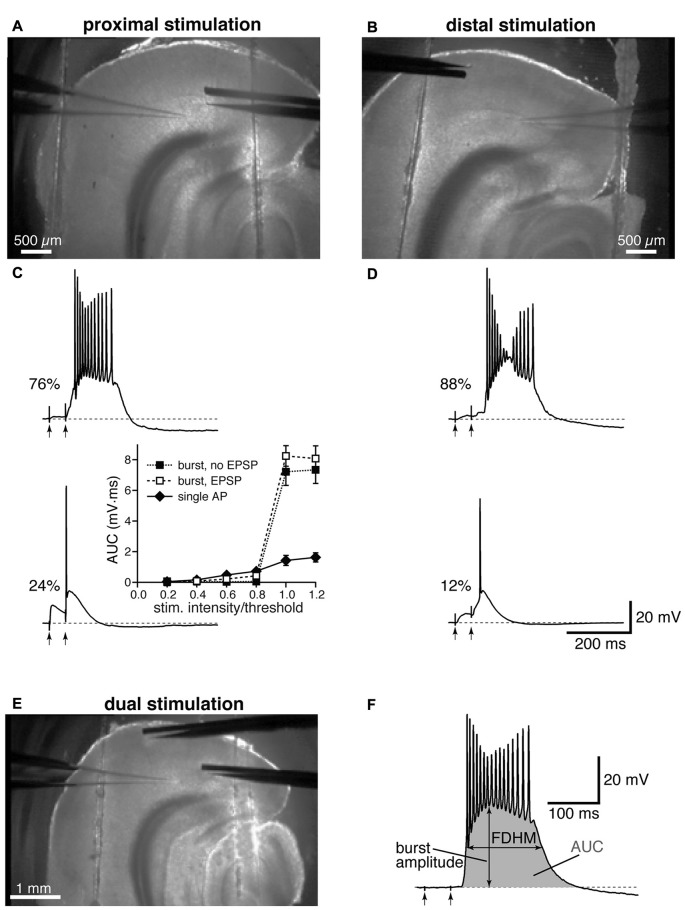
Experimental configuration and analysis details. Top: low-magnification image showing the locations of stimulation and recording electrodes for proximal **(A)** and distal **(B)** electrical stimulation. Middle: the percentages of neurons firing a burst or a single action potential (AP) in the presence of gabazine (12.5 μM) are reported next to example traces for proximal **(C)** and distal **(D)** stimulation. The arrows indicate the timing of the electrical stimulation. The inset in **(C)** shows average input-output curves for proximally-stimulated neurons which either showed burst firing without an appreciable subthreshold EPSP (filled squares), burst firing following an appreciable EPSP (open squares), or fired a single AP (filled diamonds; *n* = 5 per group). **(E)** Example of a dual stimulation experiment, in which the electrical stimulation of proximal and distal fibers was alternated. **(F)** Typical recording of a burst to demonstrate how the analysis was performed, with the full-duration at half-maximum (FDHM) quantified as the difference between the time points at which the membrane voltage reached half of the burst amplitude, and the area under the curve (AUC), shaded in gray, calculated as the integral of the depolarizing response.

Electrical stimulation was achieved by delivering constant current pulses to presynaptic afferent fibers though a tungsten bipolar electrode; in all experiments, two pulses were delivered with a 50 ms interval, and trials were separated by at least 15 s. The electrode was placed close to layer IV–V of the EC to stimulate fibers from the subiculum (40–100 μm from the soma, defined as proximal location, Figure 1A), or in the EC superficial layers (>400 μm from the soma, defined as distal location, Figure 1B). It is worth noting that the rise time constants of the synaptic responses to distal stimulation appeared to be remarkably slower than those to proximal stimulation (compare Figures 1C,D), as previously reported in Medinilla et al., 2013. This evidence confirms that distal and proximal stimulation activated two separate sets of excitatory synapses, where the kinetics of the distal inputs would be subject to more dendritic filtering than those of proximal ones (Rall, 1967). In a subset of experiments, the stimulation of proximal and distal fibers was conducted concurrently, by alternating proximal and distal stimulations, and the postsynaptic responses were recorded from the same cell (Figure 1E). At the end of each recording, a picture was taken using a low magnification objective that allows a view of the whole slice to verify the positioning of the recording and the stimulating electrodes (see Figure 1). If the patch-clamp electrode was not placed in the medial portion of layer V, below the lamina disseccans, the recording was discarded and the results not included in the analysis.

Experiments were performed in the presence of the GABA_A_ receptor antagonist gabazine (12.5 μM; Abcam, Cambridge, MA, USA) to induce hyperexcitability (Medinilla et al., 2013). The intensity of stimulation to the fibers was adjusted by 1–5 μA increments until the minimal intensity needed to evoke a suprathreshold response in the form of an AP, or a burst (whichever came first) was reached. Once the suprathreshold response was obtained, the intensity of stimulation was adjusted up and down in 0.1–0.5 μA increments to find the exact threshold intensity. We found that this value was reproducible to a 1-μA resolution. The same intensity was then maintained to confirm the occurrence of the response for 5 min before perfusing a pharmacologic agent. The experiments were continued only if a burst was initiated in at least one of the dendritic locations, and a pharmacological agent was applied and its effects were assessed after at least 5 min of drug perfusion. Stimulus intensity was then readjusted to obtain a suprathreshold response, as specified for each pharmacological agent in the “Results” Section.

NiCl_2_, nimodipine and FFA were purchased from Sigma-Aldrich (St. Louis, MO, USA); SKF 96365 was purchased from Abcam and 9-phenanthrol from Tocris/R&D Systems (Minneapolis, MN, USA). D,L-2-amino-5-phosphonovaleric acid (APV) was provided by the NIMH’s Chemical Synthesis and Drug Supply Program. Most drugs were added to the extracellular solution from a 1000× stock to achieve the final concentration (the stock for 9-phenanthrol was 500× due to solubility issues). APV, NiCl_2_ and SKF 96365 were dissolved in water; nimodipine and FFA were dissolved in DMSO (final concentration of DMSO in the extracellular solution was ≤0.1%); 9-phenanthrol was dissolved in ethanol. The nimodipine and 9-phenanthrol stocks were prepared daily; nimodipine was kept in the dark throughout the preparation and experiment to avoid oxidation.

In a set of experiments, we locally applied FFA (100 μM) around the proximal dendrite of the examined neurons by using a pressure ejection system (Picospritzer; Parker Hannifin, Pine Brook, NJ, USA) connected to a pipette ruptured to obtain a diameter of 5–10 μm; pressure was applied in a range of 0.2–0.6 psi. From previous work, this experimental configuration yielded a local perfusion along a dendritic length of ~200 μm in the vicinity of the tip of the pipette (Gasparini, 2011).

### Data Analysis

The responses to synaptic stimulation were quantified using two parameters, the area under the curve (AUC) and the full-duration at half-maximum (FDHM), utilizing routines written in the Igor Pro software (WaveMetrics, Lake Oswego, OR, USA). The AUC was quantified by calculating the integral of the depolarizing response. The FDHM was quantified as the interval between the time points at which the membrane voltage reached half of the burst amplitude (see Figure 1F). The burst amplitude was quantified as the difference between the most depolarized point during the plateau (excluding APs or AP-like deflections) and the resting membrane potential (see Figure 1F).

Data are reported as means ± SEM. Statistical comparisons were performed using one-way ANOVA (to compare all the pretreatment groups) and paired *t*-test (to compare the effect of pharmacological agents on the burst parameters). In addition, the location-dependence of the pharmacological treatments was assessed by using two-way repeated-measures ANOVA (for those pharmacological treatments where proximal and distal stimulations where performed on the same neuron), or two-way mixed ANOVA (for those pharmacological treatments where proximal and distal stimulations where performed on different neurons). The dependent variable was normally distributed for every category of the independent variable for all experimental groups except FFA, where data were converted by applying a logarithmic transformation. Means were considered to be significantly different when *P* < 0.05.

Since there appeared to be some variability in the average control values of the AUC and FDHM, we conducted a one-way ANOVA to determine if these parameters were different for the groups that were subsequently tested with the various drugs (APV, nimodipine, NiCl_2_, FFA, SKF 96565 and 9-phenanthrol). We found that the differences among the control groups were not statistically different for any of the parameters considered (*F*_(5,48)_ = 0.290, *P* = 0.916 for the proximal AUC; Welch’s *F*_(5,19.637)_ = 1.824, *P* = 0.155 for the proximal FDHM; Welch’s *F*_(5,19.979)_ = 0.485, *P* = 0.734 for the distal AUC; Welch’s *F*_(5,19.814)_ = 2.054, *P* = 0.115 for the distal FDHM).

## Results

Layer V neurons in the medial entorhinal cortex receive excitatory glutamatergic inputs at both proximal and distal dendritic locations (Jones, 1987; Canto et al., 2012; Medinilla et al., 2013). Our lab has previously shown that, in the presence of GABA_A_ receptor antagonists, the electrical stimulation of afferent fibers to layer V medial entorhinal neurons induces a form of hyperexcitability, evidenced by the initiation of bursts (Medinilla et al., 2013). The initial observation was that this hyperexcitability was more pronounced for distal synapses, where it was observed in the vast majority of the recordings, whereas it was only observed in a subset of the neurons with proximal synaptic stimulation (see Medinilla et al., 2013). This differential incidence led us to hypothesize that there might be different mechanisms underlying the generation of hyperexcitability of the EC layer V neurons at proximal and distal dendritic locations.

In the present study, we used the GABA_A_ antagonist gabazine (12.5 μM) to block synaptic inhibition and examined the response to the stimulation of proximal or distal afferent fibers for a total of 200 experimental observations (83 stimulated distally and 117 proximally); in some experiments (24 instances), the same neuron was stimulated by alternately activating proximal and distal presynaptic afferent fibers. In each experiment, two pulses were delivered to the stimulating electrode with a 50 ms-interval. The intensity of stimulation was systematically increased until a suprathreshold response was evoked in the soma. The suprathreshold responses were either in the form of a single AP, which rose on top of the excitatory synaptic response, or a burst of high frequency APs, which initiated either during the rising phase, following the rising phase, or at the end of the second EPSP, and outlasted the electrical stimulation by hundreds of milliseconds (Figures 1C,D). As in Medinilla et al. (2013), we defined these bursts as a form of hyperexcitability. Mechanisms previously implicated in epileptogenic activity include intrinsic (Mantegazza et al., 2010) and synaptic mechanisms, including recurrent excitatory activity (Wong et al., 1986). It is therefore possible that the hyperexcitability we observe is due to a combination of these mechanisms, with the reverberating network activity caused in this case by recurrent excitation of entorhinal layer V neurons (Dhillon and Jones, 2000), that is no longer effectively suppressed by inhibitory interneurons in the presence of gabazine. Confirming our previous results (Medinilla et al., 2013), this form of hyperexcitability was more frequently observed for distal stimulation (Figure 1D), where 73 out of 83 neurons exhibited hyperexcitability (88% of the sampled neurons). In comparison 89 out of 117 neurons exhibited burst firing when stimulated proximally (76% of the sampled neurons, Figure 1C). The two firing modes (single AP and burst) appear to be distinct, since notable increases in the intensity of stimulation did not cause the neurons to switch between the two modes. The inset in Figure 1C shows that the AUC (see “Materials and Methods” Section) increased only slightly upon reaching AP threshold for the neurons firing a single AP, whereas there was a major increase for the neurons that fired in bursts, regardless of whether the burst was preceded by an appreciable subthreshold depolarization. In six neurons that were firing one single AP upon proximal stimulation, increasing the intensity of stimulation above firing threshold (up to 1.05−1.3× threshold) did not shift the firing mode to burst; the same was found in four neurons stimulated distally, where the intensity of stimulation was increased up to 1.05−1.7× threshold.

Because the bursts occur on top of a long-lasting depolarizing plateau, we hypothesized that Ca^2+^-dependent mechanisms might contribute to this phenomenon and therefore investigated the relative contribution of these mechanisms. To compare the features of the bursts, we quantified two parameters: the AUC and the FDHM (see “Materials and Methods” Section). In some instances, the pharmacological agents appeared to change the stimulation intensity required for the burst; in each experiment we re-adjusted the intensity of stimulation to the lowest one that was able to initiate a suprathreshold response. For most pharmacological agents, this response was again in the form of a burst; the adjustment in stimulation intensity is indicated as an average for each pharmacological treatment below.

**Figure 2:**
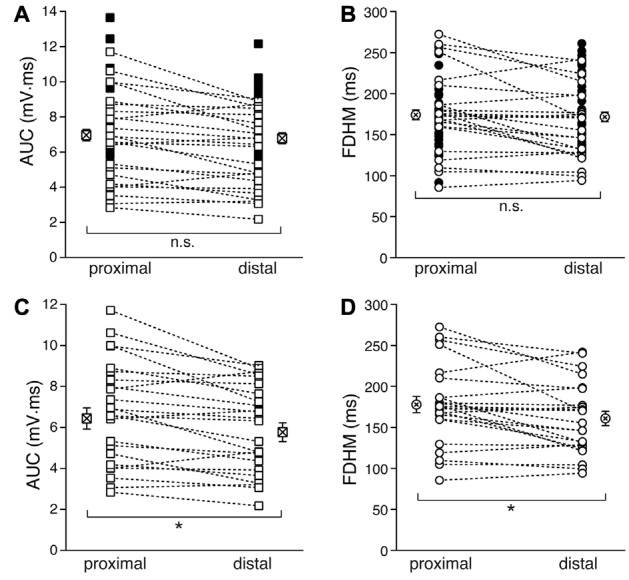
Comparison of the burst features between proximal and distal stimulation. Top: the burst AUC **(A)** and the FDHM **(B)** appeared not to be significantly different between proximal and distal stimulations when the parameters for all the neurons with at least one stimulation location were compared (unpaired *t*-test). In these panels, open symbols connected by a dotted line represent values obtained from alternate stimulation of proximal and distal afferents to the same neuron, whereas filled symbols represent values obtained from neurons in which only one of the locations was stimulated. Bottom: when the comparisons were made by paired *t*-test in those neurons that were stimulated both proximally and distally, the AUC **(C)** and the FDHM **(D)** were significantly larger for bursts evoked by proximal stimulation than distal. Asterisks indicate significant difference.

We first compared the properties of the bursts evoked by proximal and distal stimulation (Figure 2). In Figures 2A,B, the filled symbols are from neurons in which only one dendritic location was stimulated, whereas the open symbols refer to recordings from proximal and distal stimulations obtained in the same neurons. When considering the parameters for all the neurons with at least one stimulation location, the mean AUC was 7.0 ± 0.3 mV·ms (*n* = 54, Figure 2A) for proximal stimulation, which was not significantly different from that recorded for distal stimulation (6.8 ± 0.3 mV·ms, *n* = 54, *p* = 0.916, unpaired *t*-test). The mean FDHM for proximal stimulation was 174.1 ± 6.1 ms (*n* = 54, Figure 2B), which was not significantly different from that recorded for distal stimulation (171.6 ± 5.8, *n* = 54, *p* = 0.863, unpaired *t*-test). However, when we compared the AUC measured in those neurons that were stimulated both proximally and distally (*n* = 24), we found that the mean AUC for proximal stimulation was significantly larger (6.4 ± 0.5 mV·ms) than that for distal stimulation (5.8 ± 0.5 mV·ms; *t*_(23)_ = 3.732, *p* = 0.001, paired *t-test*, Figure 2C), as was the FDHM (177.9 ± 10.0 ms for proximal stimulation vs. 160.9 ± 8.7 ms; *t*_(23)_ = 3.175, *p* = 0.004, paired *t-test*, Figure 2D).

**Figure 3:**
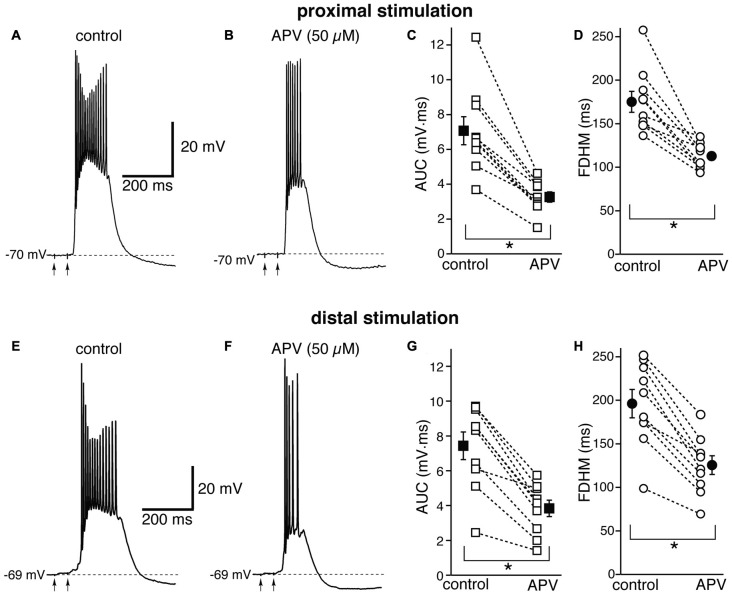
The glutamatergic NMDA receptor antagonist D,L-2-amino-5-phosphonovaleric acid (APV) reduces the duration and area of the bursts by a similar proportion in EC layer V neurons, at both proximal and distal locations. Top, representative trace of a burst recorded upon stimulation of proximal afferent fibers in control conditions **(A)** and in the presence of APV (50 μM, **B**). The arrows indicate the timing of the electrical stimulation. APV reduced the AUC **(C)** by 53 ± 3% and the FDHM **(D)** by 34 ± 4% in *n* = 10 neurons. Bottom, representative trace of a burst recorded upon stimulation of distal afferent fibers in control conditions **(E)** and in the presence of APV (50 μM, **F**). APV reduced the AUC **(G)** by 48 ± 4%, and the FDHM **(H)** by 36 ± 2% in *n* = 10 neurons. Asterisks indicate significant difference between control and APV treatment. There was no statistically significant interaction between the APV treatment and location (two-way mixed ANOVA). The traces in **(A,B)** and **(E,F)** were recorded from two separate neurons.

We first tested the contribution of NMDA glutamatergic receptors by perfusing the antagonist DL-2-Amino-5-phosphonovaleric acid (DL-APV, 50 μM, Figure 3). The inhibition of NMDA receptors significantly reduced both the duration of the plateau and the AUC. With proximal stimulation (Figure 3, top), the mean AUC value in 10 neurons decreased from 7.1 ± 0.8 mV·ms in control conditions to 3.3 ± 0.3 mV·ms in the presence of APV (a reduction by 53 ± 3%; *t*_(9)_ = 7.474, *p* < 0.005). The mean FDHM were 175.1 ± 12.0 ms and 112.8 ± 4.7 ms in control conditions and APV, respectively (a reduction by 34 ± 4%; *t*_(9)_ = 6.847, *p* < 0.005). The intensity of stimulation was increased by 5 ± 5% with respect to control conditions to initiate a suprathreshold response in the presence of APV. With distal stimulation (Figure 3, bottom), the mean value of AUC in 10 neurons was reduced by APV by 48 ± 4% (from 7.4 ± 0.8 mV·ms in control conditions to 3.8 ± 0.5 mV·ms during APV perfusion; *t*_(9)_ = 7.907, *p* < 0.005), and the mean value of FDHM was reduced by 36 ± 2% (from 196.1 ± 16.3 ms to 125.5 ± 10.7 ms in control conditions and in the presence of APV, respectively; *t*_(9)_ = 9.098, *p* < 0.005). The intensity of stimulation was increased by 9 ± 6% with respect to control conditions to initiate a suprathreshold response in the presence of APV. There was no statistically significant interaction between the APV treatment and location for either the AUC (*F*_(1,18)_ = 0.058, *p* = 0.812) or the FDHM (*F*_(1,18)_ = 0.742, *p* = 0.4, two-way mixed ANOVA). These data suggest that the activation of NMDA receptors strongly contributes to the plateau underlying bursts of hyperexcitability at both distal and proximal synapses.

**Figure 4:**
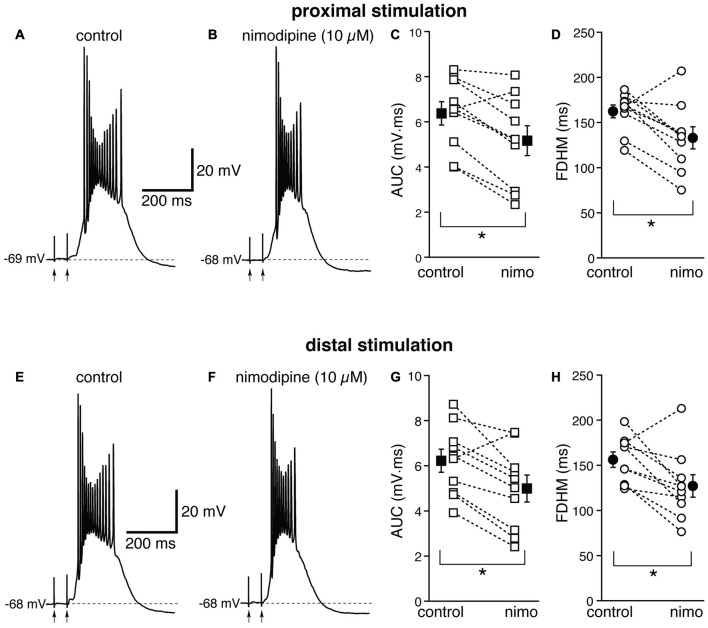
The L-type Ca^2+^ channel blocker nimodipine reduces the bursts in EC layer V neurons, at both proximal and distal locations. Top, representative trace of a burst recorded upon stimulation of proximal afferent fibers in control conditions **(A)** and in the presence of nimodipine (10 μM, **B**). Nimodipine reduced the AUC **(C)** by 21 ± 6%, and the FDHM **(D)** by 19 ± 6% in *n* = 10 neurons. Bottom, representative trace of a burst recorded upon stimulation of distal afferent fibers in control conditions **(E)** and in the presence of nimodipine **(F)**. Nimodipine reduced both the AUC **(G)** by 21 ± 6%, and the FDHM **(H)** by 19 ± 6% in *n* = 10 neurons. Asterisks indicate significant difference between control and nimodipine treatment. There was no statistically significant interaction between the nimodipine treatment and location (two-way repeated measures ANOVA). The traces in **(A,B)** and **(E,F)** were recorded from the same neuron.

Next, we investigated the contribution of various voltage-dependent Ca^2+^ currents. We first used nimodipine (10 μM) to block L-type Ca^2+^ channels (Figure 4). The blockade of L-type Ca^2+^ channels by nimodipine significantly reduced both the duration of the plateau and the AUC at both proximal and distal locations. With proximal stimulation (Figure 4, top), the mean values of AUC in 10 neurons decreased from 6.4 ± 0.5 mV·ms in control conditions to 5.2 ± 0.7 mV·ms in the presence of nimodipine (a reduction by 21 ± 6%; *t*_(9)_ = 4.325, *p* = 0.002); the mean FDHM was 162.4 ± 7.2 ms and 133.1 ± 12.3 ms in control conditions and nimodipine, respectively (a reduction by 19 ± 6%; *t*_(9)_ = 3.214, *p* = 0.011). The intensity of stimulation was increased by 5 ± 6% with respect to control conditions to initiate a suprathreshold response in the presence of nimodipine. With distal stimulation (Figure 4, bottom), the mean value of AUC in ten neurons was reduced by nimodipine by 21 ± 6% (6.2 ± 0.6 mV·ms in control conditions and 5.0 ± 0.6 mV·ms during nimodipine perfusion; *t*_(9)_ = 3.770, *p* = 0.004). The mean value of FDHM was reduced by 19 ± 6% (156.3 ± 8.4 ms vs. 127.1 ± 12.5 ms in control conditions and in the presence of nimodipine, respectively; *t*_(9)_ = 2.931, *p* = 0.017). The intensity of stimulation was increased by 2 ± 7% with respect to control conditions to initiate a suprathreshold response in the presence of nimodipine. There was no statistically significant interaction between the nimodipine treatment and location for either the AUC (*F*_(1,9)_ = 0.031, *p* = 0.864) or the FDHM (*F*_(1,9)_ = 0.001, *p* = 0.977, two-way repeated measures ANOVA).

These data suggest that the activation of L-type Ca^2+^ channels also contributes to the plateau underlying hyperexcitable bursts at both distal and proximal synapses, although to a lesser extent than NMDA glutamatergic receptors.

**Figure 5:**
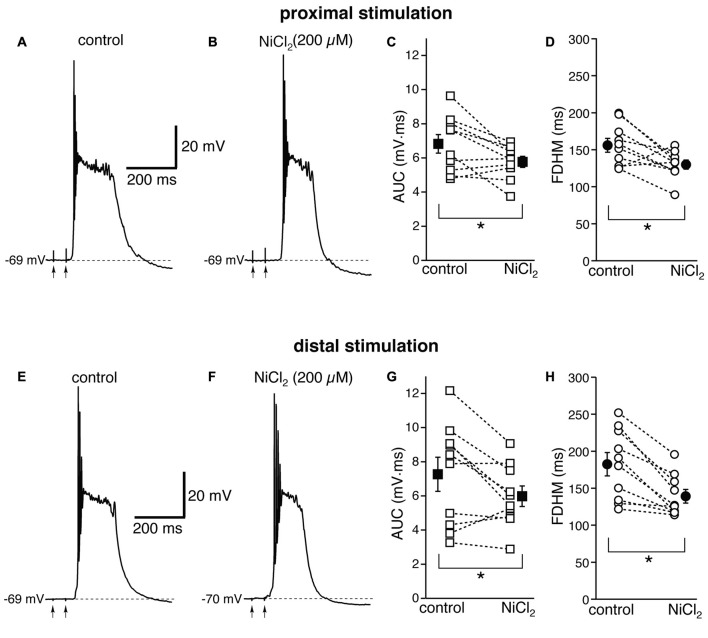
NiCl_2_, at a concentration that blocks both R- and T-type Ca^2+^ channels, reduces the depolarizing plateaus and bursts at proximal and distal synapses. Top, representative trace of a burst recorded upon stimulation of proximal afferent fibers in control conditions **(A)** and in the presence of NiCl_2_ (200 μM, **B**). NiCl_2_ reduced the AUC **(C)** by 13 ± 6% and the FDHM **(D)** 15 ± 6% in *n* = 10 neurons. Bottom, representative trace of a burst recorded upon stimulation of distal afferent fibers in control conditions **(E)** and in the presence of NiCl_2_ **(F)**. NiCl_2_ reduced the AUC **(G)** by 22 ± 4% and the FDHM **(H)** by 12 ± 8% in *n* = 10 neurons. Asterisks indicate significant difference between control and NiCl_2_ treatment. There was no statistically significant interaction between the NiCl_2_ treatment and location (two-way mixed ANOVA). The traces in **(A,B)** and **(E,F)** were recorded from two separate neurons.

We then investigated the possible contribution of other subtypes of voltage-dependent Ca^2+^ channels, in particular R-type Ca^2+^ channels, which have been shown to contribute to bursting in CA1 pyramidal neurons (Magee and Carruth, 1999). To this end, we used NiCl_2_, at a concentration of 200 μM (Figure 5), which was shown to be maximally effective in reducing the after-depolarization that can underlie burst firing in CA1 pyramidal neurons (Metz et al., 2005). This concentration of NiCl_2_ has also been shown to block T-type Ca^2+^ channels as well (Fox et al., 1987; Bean, 1989). At proximal synapses (Figure 5, top), NiCl_2_ reduced the average AUC in 10 neurons by 13 ± 6% (from 6.8 ± 0.5 mV·ms in control conditions to 5.8 ± 0.3 mV·ms in the presence of NiCl_2_; *t*_(9)_ = 2.622, *p* = 0.028), and the FDHM by 15 ± 6% (from 156.0 ± 9.3 ms in control conditions to 129.9 ± 6.1 ms in the presence of NiCl_2_; *t*_(9)_ = 2.778, *p* = 0.021). The intensity of stimulation was increased by 28 ± 5% with respect to control conditions to initiate a suprathreshold response in the presence of NiCl_2_. On the other hand, at distal synapses (Figure 5, bottom) NiCl_2_ decreased the FDHM by 22 ± 4% (from 182.4 ± 15.7 ms in control conditions to 139.2 ± 9.2 ms in the presence of NiCl_2_ in 10 neurons; *t*_(9)_ = 4.758, *p* = 0.001), and the AUC by 12 ± 8% (7.3 ± 1.0 mV·ms in control conditions vs. 6.0 ± 0.6 mV·ms in the presence of NiCl_2_; *t*_(9)_ = 2.332, *p* = 0.045). The intensity of stimulation was increased by 30 ± 9% with respect to control conditions to initiate a suprathreshold response in the presence of NiCl_2._ There was no statistically significant interaction between the NiCl_2_ treatment and location for either the AUC (*F*_(1,18)_ = 0.154, *p* = 0.700) or the FDHM (*F*_(1,18)_ = 2.044, *p* = 0.17, two-way mixed ANOVA). These data suggest that R- and T-type Ca^2+^ channels also contribute to the plateaus underlying hyperexcitability at proximal and distal synapses.

**Figure 6:**
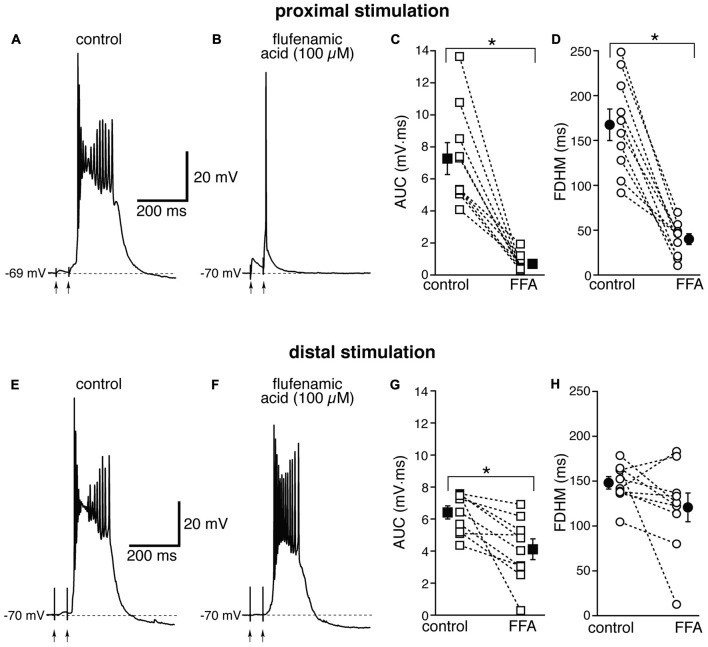
The effect of flufenamic acid (FFA) on the depolarizing plateau and hyperexcitability differs depending on the location of the stimulation. Top, representative trace of the response elicited by synaptic stimulation in control conditions **(A)** and in the presence of FFA (100 μM, **B**) for proximal synaptic stimulation. Strikingly, the perfusion of FFA changes the response from a burst into a single AP. This fact is reflected in the marked average decrease of the AUC (**C**, by 88 ± 4%) and the FDHM (**D**, by 74 ± 5%) in *n* = 10 neurons. Bottom, representative trace of a burst recorded upon stimulation of distal afferent fibers in control conditions **(E)** and in the presence of FFA **(F)**. FFA switched the response from a burst to a single AP in only one neuron. In addition, on average FFA reduced the AUC **(G)** by only 35 ± 9%, and the FDHM **(H)** by only 17 ± 11% in *n* = 10 neurons. Asterisks indicate significant difference between control and FFA treatment. There was a statistically significant interaction between the FFA treatment and location for both the AUC (*p* < 0.0005) and the FDHM (*p* = 0.002, two-way mixed ANOVA). The traces in **(A,B)** and **(E,F)** were recorded from two separate neurons.

The Ca^2+^-activated non-selective cation current (*I*_CAN_), mediated by TRP channels (Ramsey et al., 2006), has been reported to be involved in the generation of NMDA-dependent bursting activity in dopaminergic neurons of the substantia nigra pars compacta (Mrejeru et al., 2011). In entorhinal layer V neurons, a subtype of TRP channels have been shown to be involved in the generation of graded persistent activity in the presence of cholinergic agonists (Egorov et al., 2002; Zhang et al., 2011). For this reason, we tested the effect on the bursts of a broad-spectrum, non-competitive antagonist for TRP channels (Clapham, 2007), FFA, at a concentration of 100 μM. In contrast to all the other drugs we tested, the effect of FFA was strikingly different at proximal and distal synapses. For proximal stimulation, FFA abolished burst firing and shifted the suprathreshold response to a single AP (compare Figures 6A,B) in all 10 neurons tested. As a consequence, FFA markedly decreased both the average AUC (by 88 ± 4%, from 7.3 ± 1.0 mV·ms in control conditions to 0.7 ± 0.2 mV·ms in the presence of FFA; *t*_(9)_ = 7.108, *p* < 0.0005) and the FDHM (by 74 ± 5%, from 167.4 ± 17.6 ms in control conditions to 40.1 ± 6.2 ms in the presence of FFA; *t*_(9)_ = 8.369, *p* < 0.0005). In response to proximal stimulation, the AP threshold was not significantly different (*t*_(9)_ = 0.586, *p* = 0.572), in the presence of FFA (−47.8 ± 1.2 mV) with respect to control conditions (−47.0 ± 0.7 mV). In four of these neurons, we increased the intensity of stimulation above firing threshold (up to 1.1−1.5× threshold) and a single AP was always observed in the presence of FFA. The intensity of stimulation was increased by 95 ± 21% with respect to control conditions to initiate a suprathreshold response in the presence of FFA. In contrast, for distal stimulation, only 1 out of 10 neurons switched from firing in bursts to firing a single AP. The intensity of stimulation was increased by 57 ± 20% with respect to control conditions to initiate a suprathreshold response in the presence of FFA. On average, for distal stimulation FFA decreased the AUC by 35 ± 9%, (from 6.4 ± 0.4 mV·ms in control conditions to 4.1 ± 0.6 mV·ms in the presence of FFA;* t*_(9)_ = 3.714, *p* = 0.005), and the FDHM by 17 ± 11% (from 148.1 ± 7.0 ms in control conditions to 120.8 ± 16.1 ms in the presence of FFA; *t*_(9)_ = 1.906, *p* = 0.089). In response to distal stimulation, the AP threshold was not significantly (*t*_(9)_ = −1.297, *p* = 0.227) different in the presence of FFA (−46.3 ± 0.9 mV) with respect to control conditions (−46.9 ± 0.9 mV). There was a statistically significant interaction between the FFA treatment and location for both the AUC (*F*_(1,18)_ = 27.916, *p* < 0.0005) and the FDHM (*F*_(1,18)_ = 13.306, *p* = 0.002, two-way mixed ANOVA). It should also be noted that in the only experiment that was performed with dual stimulation for this pharmacological treatment, proximal stimulation shifted to a single AP, whereas a burst was still observed for distal stimulation in the presence of FFA, confirming the overall pattern.

**Figure 7:**
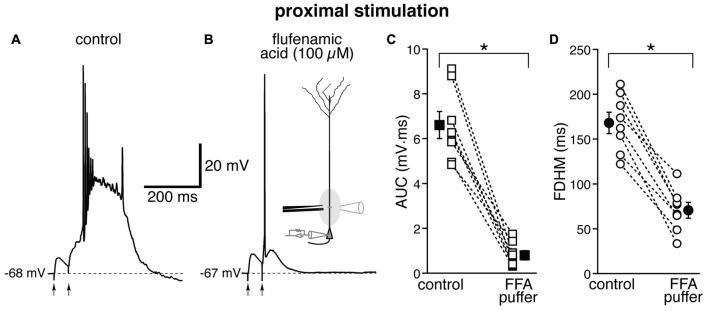
Local perfusion of FFA abolishes burst firing for proximal stimulation. Representative trace of the response elicited by proximal synaptic stimulation in control conditions **(A)** and when FFA (100 μM, **B**) was locally applied through a ruptured pipette connected to a picospritzer; the inset in **(B)** shows a diagram of the experimental configuration. In seven out of eight neurons, local perfusion of FFA changed the postsynaptic suprathreshold response from a burst into a single AP. This effect of FFA is reflected in the marked average decrease of the AUC (**C**, by 87 ± 4%) and the FDHM (**D**, by 58 ± 4%). Asterisks indicate significant difference between control conditions and local FFA perfusion.

The location-dependence of the effect of FFA we observed could be due to a differential expression of TRP channels at proximal and distal dendritic locations in entorhinal layer V neurons, or to a generalized effect on the excitatory circuits. In particular, FFA at higher concentrations has been shown to reduce the activity of voltage-dependent Na^+^ channels in hippocampal CA1 neurons (Yau et al., 2010). By this mechanism, bath-applied FFA could cause a generalized decrease in excitability among all neurons in the slice, and therefore reduce or eliminate the recurrent excitatory activity, which we think is partially responsible for the burst activity we observe. In order to differentiate between these two hypotheses, we used a local application of FFA (100 μM, Figure 7) at proximal locations, where we have observed the largest effect under bath application (see Figure 6). From previous experiments (Gasparini, 2011), we have found that this configuration achieves a local perfusion of ~200 μm in the vicinity of the puffer pipette. The local perfusion of FFA abolished burst firing and shifted the suprathreshold response to a single AP in seven of the eight neurons tested (Figures 7A,B). As a consequence, FFA markedly decreased both the average AUC (by 87 ± 4%, from 6.1 ± 0.6 mV·ms in control conditions to 0.8 ± 0.2 mV·ms in the presence of FFA; *t*_(7)_ = 9.111, *p* < 0.0005), and the FDHM (by 58 ± 4%, from 168.1 ± 12.0 ms in control conditions to 70.6 ± 8.9 ms in the presence of FFA; *t*_(7)_ = 10.258, *p* < 0.0005). The intensity of stimulation was increased by 123 ± 25% with respect to control conditions to initiate a suprathreshold response in the presence of FFA. The AP threshold was slightly, but not significantly (*t*_(7)_ = 1.682, *p* = 0.137), more hyperpolarized in the presence of FFA (−46.6 ± 1.1 mV) than under control conditions (−45.5 ± 0.7 mV). We also measured the threshold of APs initiated by a 2 ms-depolarizing current injection at the soma. This AP threshold also was not significantly different (*t*_(5)_ = 0.181, *p* = 0.863) when measured under control conditions (−52.2 ± 0.5 mV) and during local perfusion of FFA (−52.3 ± 0.3 mV, *n* = 6). These data point towards a main effect of FFA at the proximal dendrite of the postsynaptic neurons, rather than a generalized decrease in excitability in the whole slice, in eliminating the burst activity initiated by proximal stimulation in entorhinal layer V neurons.

We further investigated the possible mechanisms underlying the effect of FFA by employing selective antagonists for the two most likely TRP sub-families, canonical TRP (TRPC) or melastatin TRP (TRPM), which are both blocked by FFA at a concentration of 100 μM (Guinamard et al., 2013).

**Figure 8:**
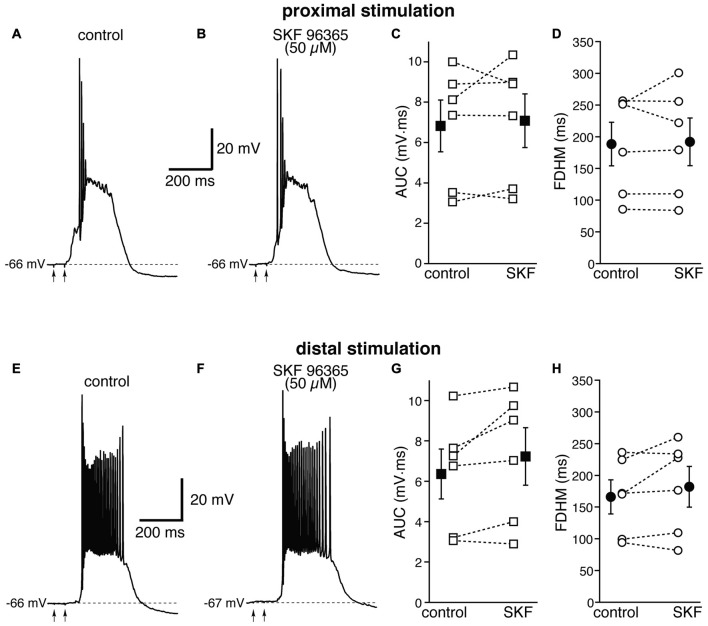
The transient receptor potential canonical (TRPC) channel blocker SKF 96365 does not affect the bursts in EC layer V neurons, at either proximal or distal locations. Top, representative trace of a burst recorded upon stimulation of proximal afferent fibers in control conditions **(A)** and in the presence of SKF 96365 (50 μM, **B**). SKF 96365 did not significantly affect either the AUC **(C)**, or the FDHM **(D)** in *n* = 6 neurons. Bottom, representative trace of a burst recorded upon stimulation of distal afferent fibers in control conditions **(E)** and in the presence of SKF 96365 **(F)**. SKF 96365 did not significantly affect either the AUC **(G)**, or the FDHM **(H)** in *n* = 6 neurons. The traces in **(A,B)** and **(E,F)** were recorded from different neurons.

We first used SKF 96365 (50 μM), an antagonist for TRPC channels (Vazquez et al., 2004, Figure 8). This pharmacological agent has been shown to inhibit carbachol-evoked plateau potentials and persistent firing in layer V entorhinal neurons (Zhang et al., 2011). At proximal synapses, SKF 96365 caused a small, not significant increase (by 5 ± 7%, *t*_(5)_ = −0.554, *p* = 0.604) in the average AUC in six neurons, from 6.8 ± 1.3 mV·ms in control conditions to 7.1 ± 1.3 mV·ms in the presence of SKF, and in the average FDHM (by 1 ± 4%, *t*_(5)_ = −0.339, *p* = 0.748), from 188.5 ± 34.4 ms in control conditions to 191.9 ± 37.6 ms in the presence of SKF (Figure 8, top). The intensity of stimulation was decreased by 10 ± 4% with respect to control conditions to initiate a suprathreshold response in the presence of SKF. At distal synapses SKF 96365 induced a small, not significant increase in the average FDHM in six neurons (by 8 ± 7%, *t*_(5)_ = −1.461, *p* = 0.204) from 166.0 ± 26.8 ms in control conditions to 181.5 ± 32.3 ms in the presence of SKF, and in the average AUC (by 13 ± 7%, *t*_(5)_ = −2.241, *p* = 0.075) from 6.4 ± 1.2 mV·ms in control conditions vs. 7.2 ± 1.4 mV·ms in the presence of SKF (Figure 8, bottom). The intensity of stimulation was increased by 1 ± 10% with respect to control conditions to initiate a suprathreshold response in the presence of SKF. There was no statistically significant interaction between the SKF 96365 treatment and location for either the AUC (*F*_(1,10)_ = 1.054, *p* = 0.329) or the FDHM (*F*_(1,10)_ = 0.668, *p* = 0.433, two-way mixed ANOVA).

**Figure 9:**
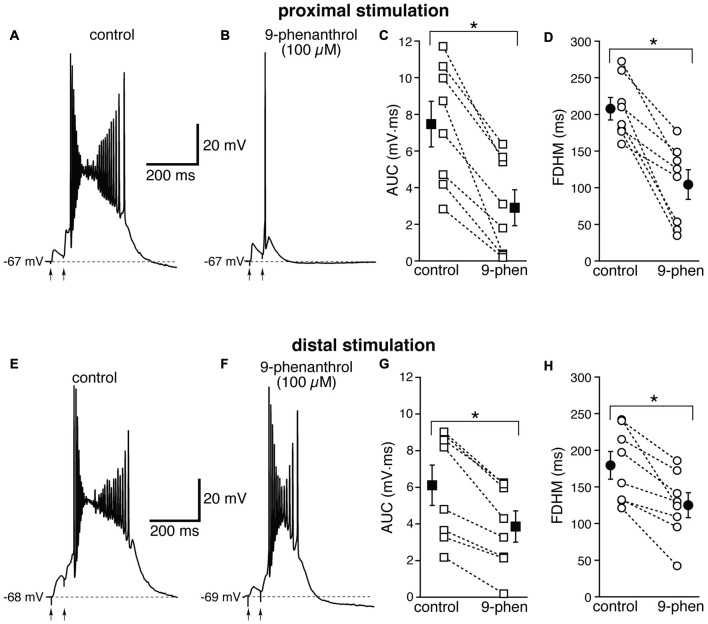
The effect of 9-phenanthrol on the depolarizing plateau and hyperexcitability differs depending on the location of the stimulation. Top, representative trace of the response elicited by synaptic stimulation in control conditions **(A)** and in the presence of 9-phenanthrol (100 μM, **B**). The perfusion of 9-phenanthrol changed the response from a burst into a single AP in four out of eight neurons. This fact is reflected in the striking reduction in the average AUC (by 67 ± 9%, **C**) and the FDHM (by 50 ± 9%, **D**). Bottom, representative trace of a burst recorded upon stimulation of distal afferent fibers in control conditions **(E)** and in the presence of 9-phenanthrol **(F)**. 9-phenanthrol switched the response from a burst to a single AP in only one neuron out of eight. In addition, 9-phenanthrol significantly reduced the average AUC (by 42 ± 8%, **G**) and the FDHM (by 31 ± 7%, **H**) in *n* = 8 neurons. Asterisks indicate significant difference between control and 9-phenanthrol. There was a statistically significant interaction between the 9-phenanthrol treatment and location for both the AUC (*p* = 0.001) and the FDHM (*p* = 0.009, two-way repeated measures ANOVA). The traces in **(A,B)** and **(E,F)** were recorded from the same neuron.

We therefore examined the effect of 9-phenanthrol, a selective inhibitor for TRPM channels, in particular TRPM4 (Grand et al., 2008), which has been shown to abolish NMDA-induced bursting in dopamine neurons of the substantia nigra pars compacta (Mrejeru et al., 2011). As in the case of FFA, 9-phenanthrol (100 μM) appeared to differentially affect the burst initiated by proximal and distal stimulations. In all these experiments, proximal and distal stimulation were performed alternately on the same neuron. For proximal stimulation, 9-phenanthrol abolished burst firing and switched the suprathreshold response from a burst to a single AP (compare Figures 9A,B) in four out of the eight neurons tested. As a consequence, 9-phenanthrol significantly decreased both the mean AUC in eight neurons by 67 ± 9% (from 7.5 ± 1.2 mV·ms in control conditions to 2.9 ± 1.0 mV·ms in the presence of 9-phenanthrol;* t*_(7)_ = 6.853, *p* < 0.0005, Figure 9C) and the FDHM by 50 ± 9% (from 207.7 ± 15.3 ms in control conditions to 104.3 ± 20.3 ms in the presence of 9-phenanthrol;* t*_(7)_ = 6.027, *p* = 0.001, Figure 9D). The AP threshold for APs generated by proximal stimulation was slightly, but not significantly (*t*_(7)_ = 2.337, *p* = 0.052) more hyperpolarized in the presence of 9-phenanthrol (−48.0 ± 1.1 mV) than under control conditions (−46.4 ± 0.6 mV). The intensity of stimulation was increased by 52 ± 14% with respect to control conditions to initiate a suprathreshold response in the presence of 9-phenanthrol. In the four neurons in which 9-phenanthrol switched the firing mode from burst into single AP, we increased the intensity of stimulation above threshold (up to 1.1−1.3× threshold) and a single AP was always observed in the presence of 9-phenanthrol. In contrast, for distal stimulation, only one out of eight neurons switched from firing in bursts to firing a single AP. On average, for distal stimulation 9-phenanthrol decreased the average AUC in eight neurons by 42 ± 8% (from 6.1 ± 1.1 mV·ms in control conditions to 3.9 ± 0.8 mV·ms in the presence of 9-phenanthrol;* t*_(7)_ = 7.046, *p* < 0.0005), and the FDHM (a reduction by 31 ± 7%, from 179.4 ± 19.0 ms in control conditions to 125.2 ± 17.0 ms in the presence of 9-phenanthrol;* t*_(7)_ = 4.882, *p* = 0.002). The threshold for APs initiated by distal stimulation was not significantly different (*t*_(7)_ = 0.500, *p* = 0.632) in the presence of 9-phenanthrol (−46.6 ± 1.0 mV) with respect to control conditions (−46.9 ± 0.7 mV). The intensity of stimulation was increased by 90 ± 50% with respect to control conditions to initiate a suprathreshold response in the presence of 9-phenanthrol. There was a statistically significant interaction between the 9-phenanthrol treatment and location for both the AUC (*F*_(1,7)_ = 29.965, *p* = 0.001) and the FDHM (*F*_(1,7)_ = 12.840, *p* = 0.009, two-way repeated measures ANOVA). It is also worth noting that, of the four neurons in which proximal stimulation switched from burst to single AP firing, only one switched to a single AP for the distal stimulation as well, whereas three kept firing in bursts upon distal stimulation. Since 9-phenanthrol was dissolved from a 500× stock in ethanol, we performed control experiments to evaluate a possible effect of the vehicle on the burst parameters in the case of proximal stimulation (data not shown). In *n* = 7 experiments, we found that this concentration of ethanol (0.2%) reduced the FDHM by 6 ± 3% and the AUC by 11 ± 3%. In addition, three out of four neurons, stimulated proximally and exposed to 0.2% ethanol as a control, switched from burst firing to single AP when subsequently perfused with 9-phenanthrol. In these four neurons, the AUC decreased by 90 ± 1% and the FDHM decreased by 75 ± 1% with respect to the ethanol control, suggesting that ethanol alone was not responsible for the effect observed in the presence of 9-phenanthrol. Altogether, these data suggest that *I*_CAN_ currents, mediated presumably by TRPM4 channels, are the major mechanism responsible for the generation of hyperexcitable bursts at proximal dendritic regions of mEC layer V neurons, whereas their contribution appears to be limited at distal dendritic locations.

## Discussion

In this work, we have characterized the differential contribution of various Ca^2+^-dependent mechanisms to the bursts initiated by stimulation of excitatory afferents to proximal or distal dendritic locations in layer V neurons of the medial entorhinal cortex, in the presence of GABA_A_ receptor antagonists. The main findings are: (1) various Ca^2+^ influx mechanisms contribute to the plateau underlying the hyperexcitable bursts, since their blockade affect, to different extents, the AUC and the duration of the bursts at proximal and distal locations; (2) FFA, whether locally or bath-applied, abolishes burst firing and switches the suprathreshold response to single APs at proximal dendritic locations, but has only a minor effect at distal locations; and (3) 9-phenanthrol, a selective blocker for TRPM channels, also converts bursts into single APs for proximal, but not distal stimulation in half of the neurons tested. These results show that, in the presence of GABA_A_ receptor antagonists, a combination of synaptic (NMDA glutamatergic receptors) and intrinsic (voltage-gated Ca^2+^ channels) mechanisms appears to activate TRP (mostly TRPM) channels. At proximal dendritic locations, the activation of TRPM channels appears to be a major mechanism for the generation of hyperexcitable bursts, since the perfusion of FFA and to a lesser extent 9-phenanthrol, switches the firing mode from a burst to a single AP, whereas at distal dendritic locations this mechanism is less significant.

We first examined the features of the hyperexcitable bursts under control conditions. In some cases, the bursts were originated without a preceding discernible synaptic depolarization (see Figures 3A, 5 for some examples), in others they occurred during the depolarizing synaptic response, either on the ascending phase of the EPSP (Figure 1C), but most often initiating during the repolarizing phase (Figure 4E) or a prolonged depolarization (see Figures 6A, 9). We hypothesize that this hyperexcitability is due to a combination of the activation of ion channels intrinsic to MEC layer V neurons and the reverberating network activity caused by the presence of a high recurrent excitation among layer V neurons (Dhillon and Jones, 2000), that is no longer effectively suppressed by inhibitory interneurons in the presence of gabazine. As such, this type of response may be initiated with a delay with respect to the electrical stimulation, but the activation of ion channels (whether ligand- or voltage-activated) in the postsynaptic neurons is essential for the occurrence of the bursts, which are abolished or significantly attenuated in the presence of blockers for Ca^2+^-dependent mechanisms. We also found that the bursts were more likely initiated by distal rather than proximal stimulation (88% of the experimental instances vs. 76%). This difference in occurrence could be due to differential dendritic mechanisms underlying burst generation, or different circuits activated at proximal and distal locations. For example, axon collaterals from layer V neurons branch and extend into the superficial layers (Burgalossi et al., 2011) where they make synapses on layer II and III neurons closing the loop from the hippocampus (Chrobak and Buzsáki, 1994), and possibly layer V neurons. These axon collaterals form part of the associational system which might contribute to the EC susceptibility to epileptogenesis (Spruston and McBain, 2006). However, we cannot exclude that the higher likelihood of distal stimulation to produce somatic bursts might be due simply to the fact that local dendritic depolarization has to be stronger at distal than at proximal locations in order to reach AP threshold at the soma, due to the higher voltage attenuation along the dendrites. This stronger synaptic depolarization would be more likely to activate NMDA glutamatergic receptors and voltage-dependent Ca^2+^ channels, therefore making the initiation of a burst more likely for distal stimulation. When we compared the burst parameters for all the neurons recorded with either proximal or distal stimulation, we did not find differences in the AUC or the FDHM. Interestingly, however, when we compared within-cell the AUC and the FDHM measured in neurons in which both proximal and distal stimulation were performed, we found that both parameters were significantly smaller for bursts initiated by distal stimulation rather than proximal. This discrepancy may arise from the amount of variability in the burst responses, such that comparisons made between, rather than within, neurons might result in overlooking the relationship between responses at the two different input locations. This result could potentially argue in favor of a dendritic location for the generation of the bursts themselves, since the width and amplitude of dendritically-originated bursts decreases along dendrites (Magee and Carruth, 1999; Larkum et al., 2001).

Our results show that synaptic mechanisms, such as the activation of NMDA glutamatergic receptors, appear to contribute to the depolarizing plateaus underlying the bursts to a larger extent than voltage-dependent Ca^2+^ channels. The blockade of NMDA glutamatergic receptors by APV (50 μM) decreased the AUC by about 50% at both proximal and distal synapses, whereas the blockade of L-type Ca^2+^ channels by nimodipine decreased the AUC by ~20% and the blockade of T-type and R-type Ca^2+^ channels by NiCl_2_ (200 μM) only reduced the AUC by ~10%. The relatively small effect of NiCl_2_ on the bursts in entorhinal layer V neurons is an interesting finding, when compared to hippocampal CA1 pyramidal neurons, where the same concentration of NiCl_2_ has been shown to be maximally effective in reducing the after-depolarization that underlies burst firing (Metz et al., 2005). In addition, in CA1 neurons, lower concentrations of NiCl_2_ (75 μM) strongly reduced the burst originated by local perfusion of 4-aminopyridine (Magee and Carruth, 1999). These comparisons lead to the conclusion that entorhinal layer V neurons and CA1 pyramidal neurons might express fundamentally different bursting mechanisms, due to a differential expression of dendritic ion channels.

Whereas the Ca^2+^ influx mechanisms described above (NMDA receptors and Ca^2+^ channels) do not appear to have a location-dependent effect, we found that the effect of FFA was highly location-dependent. FFA is a non-steroidal anti-inflammatory drug that reduces synthesis of prostaglandin and inhibits cyclo-oxygenases (Flower, 1974), and has been subsequently used as a broad-spectrum blocker for TRP channels that mediate the calcium-activated non-selective cationic current *I*_CAN_ (Partridge and Valenzuela, 2000). Since FFA shifts EC layer V neurons from burst firing to single AP for proximal but not distal stimulation, we hypothesized that TRP channels are the main mechanism responsible for the hyperexcitable burst firing at proximal synapses. Bath-applied FFA (Figure 6) could abolish the burst activity by directly affecting dendritic mechanisms in the recorded neurons, or by causing a generalized decrease of excitability, which would mostly affect the recurrent excitatory network. However, since locally applied FFA in the vicinity of the proximal dendrites of the recorded neuron abolished burst firing in seven of the eight neurons tested, we tend to conclude that the main effect of FFA is on TRP channels on the proximal dendrites of entorhinal layer V neurons. The location-dependence of the effect could be due to a differential expression of the channels along the dendrites, for which there is some previous evidence (von Bohlen Und Halbach et al., 2005).

In our investigation of the TRP channel subtypes involved in the burst response, we further focused on TRPC and TRPM channels, which are blocked by FFA (100 μM). In our experimental conditions, TRPC channels could be activated, in theory, through group I metabotropic glutamatergic receptors (mGluR) and the G_q_/phospholipase C pathway (Fowler et al., 2007), or through an increase in [Ca^2+^]_i_ entering through NMDA receptors. The first mechanism has been demonstrated in the lateral septum (Tian et al., 2013), the substantia nigra (Tozzi et al., 2003) and the hippocampus (Congar et al., 1997; Gee et al., 2003), whereas the second one has been shown in the granule cells of the olfactory bulb (Stroh et al., 2012); TRPC5 subunits, in particular, can be activated directly by an increase in intracellular Ca^2+^ (Gross et al., 2009). The increase in [Ca^2+^]_i_ through the other mechanisms we have described in this work would further potentiate TRPC5 activation (Blair et al., 2009). The expression of TRPC1 and TRPC5 has been reported in the entorhinal cortex, although at a lower density than in the hippocampus (von Bohlen Und Halbach et al., 2005). Finally, TRPC4/5 channels have been shown to underlie plateau potentials and persistent activity in the presence of cholinergic agonists in these same neurons (Zhang et al., 2011). For all these reasons, it was somehow surprising to find that the TRPC inhibitor SKF 96365 did not have any significant effects on the bursting tendency in layer V entorhinal neurons.

We found instead that the effect of FFA was closely mimicked by a blocker of TRPM4 channels, 9-phenanthrol, which abolished burst firing and shifted the suprathreshold response to single APs for proximal stimulation in half of the neurons we tested. In addition, the effect of 9-phenanthrol appeared to be location-dependent, as was the case with FFA. TRPM4/5 channels are unique among TRP channels, because they are activated directly by increases in [Ca^2+^]_i_, and they are only permeable to Na^+^ and K^+^ but do not conduct Ca^2+^ (Launay et al., 2002; Ullrich et al., 2005). The peculiarity of TRPM4 channels extend to the fact that they have a voltage-dependent behavior with a strong outward rectification, due to the fact that these channels are activated at depolarized membrane potentials, in the presence of elevated [Ca^2+^]_i_ (Nilius et al., 2003). This combined Ca^2+^- and voltage-dependence makes TRPM4 channels the perfect candidate for the hyperexcitable bursts we have characterized. Along these lines, TRPM4 channels have been shown to contribute to NMDA-mediated bursting in dopamine neurons of the substantia nigra pars compacts (Mrejeru et al., 2011), and to bursts during the inspiratory phase of the respiratory cycle in neurons in the preBötzinger complex in the ventrolateral medulla oblongata (Crowder et al., 2007; Pace et al., 2007).

In addition to TRP channels, FFA is known to affect other ion channels (for a review, see Guinamard et al., 2013). Cl^−^ channels were the first group of channels shown to be affected by FFA (Suzuki et al., 2006), but their blockade would have the opposite effect than the reduction in hyperexcitability that we have observed. More relevant in this context could be the reported reduction of depolarizing currents such as voltage-dependent Na^+^ channels (Yau et al., 2010) or NMDA glutamatergic receptors (Prisco et al., 2000) by FFA; however, several observations argue against a generalized decrease of excitability in the presence of FFA. A major effect of FFA on NMDA glutamatergic receptors can be ruled out by the fact that NMDA receptor antagonists have a similar effect at proximal and distal synapses (see Figure 3), whereas the effect of FFA is fundamentally different at the two locations. As for Na^+^ channels, a significant blockade of Na^+^ channels was obtained at higher concentrations of FFA (200 μM, Yau et al., 2010) than those used in this work. In addition, a partial reduction in the activity of Na^+^ channels would mostly affect distal synapses (Gasparini and Magee, 2006), whereas we observed the most prominent effect of FFA at proximal synapses. Moreover, in the only experiment with dual stimulation for this treatment group, in which the burst firing was observed at both proximal and distal synapses, FFA switched proximal synapses to single firing but did not change the burst firing at distal synapses. For these reasons, we tend to conclude that, in our experimental conditions, the main effect of FFA at proximal synapses is mostly on TRP channels. However, since FFA was able to abolish burst firing and shift the suprathreshold response to a single AP in all the neurons tested, whereas 9-phenanthrol did so in half of the neurons tested, it is possible that some of the non-specific effects of FFA contribute to its action.

To further elaborate about the role of TRP channels, we think that, in the presence of gabazine, their activation happens quickly downstream from the activation of mechanisms that increase [Ca^2+^]_i_, and that at proximal dendritic locations they are essential for the depolarization underlying the bursts. In the presence of TRP channel blockers, this mechanism is abolished and for this reason in most cases we do not observe this prolonged depolarization upon proximal stimulation. Since we used a suprathreshold response as a readout (either burst or single AP), the stimulation intensity in these situations had to be increased to recruit more fibers until AP threshold was reached through the activation of glutamatergic receptors. For this reason, the increase in stimulation intensity needed for a suprathreshold response was much larger in the presence of TRP channel blockers. Interestingly, though, the perfusion of these agents did not seem to significantly change the voltage threshold for the first (or only) AP. In this light, therefore, it might be more accurate to define the effect of TRP blockers as abolishing the burst activity rather than switching the firing mode from bursts to single APs. Nevertheless, we have clearly shown that TRP channels are major contributors of bursting activity at proximal synapses in entorhinal layer V neurons. It would be worth investigating whether FFA or other more specific blockers of TRP channels can affect hyperexcitable bursting activity in other brain areas, since FFA has been proposed as a possible antiepileptic tool (Schiller, 2004; Yau et al., 2010).

About one percent of American population experiences some form of epilepy; the majority of antiepileptic drugs target Na^+^ channels, GABA receptors and/or Ca^2+^ channels. Specifically, these drugs mostly enhance sodium channel inactivation, increase GABA receptor-mediated inhibition, or decrease Ca^2+^ influx to suppress membrane excitability. Our study sheds light on specific mechanisms that could underlie changes in dendritic and neuronal excitability in neurons prone to epileptogenic activity, such as entorhinal layer V neurons, and suggests TRP channels as therapeutic targets that merit deeper investigation.

## Author Contributions

SG designed the study; ECL, CLC and SG performed the experiments, analyzed the data, wrote the article and approved the final version of the manuscript. ECL and CLC contributed equally to this work.

## Conflict of Interest Statement

The authors declare that the research was conducted in the absence of any commercial or financial relationships that could be construed as a potential conflict of interest.

## References

[B1] Avoli M. D’Antuono M. Louvel J. Köhling R. Biagini G. Pumain R.. (2002). Network and pharmacological mechanisms leading to epileptiform synchronization in the limbic system *in vitro*. Prog. Neurobiol. 68, 167–207. 10.1016/s0301-0082(02)00077-1 12450487

[B2] Bean B. P. (1989). Classes of calcium channels in vertebrate cells. Annu. Rev. Physiol. 51, 367–384. 10.1146/annurev.physiol.51.1.367 2540697

[B3] Blair N. T. Kaczmarek J. S. Clapham D. E. (2009). Intracellular calcium strongly potentiates agonist-activated TRPC5 channels. J. Gen. Physiol. 133, 525–546. 10.1085/jgp.200810153 19398778 PMC2712973

[B4] Burgalossi A. Herfst L. von Heimendahl M. Förste H. Haskic K. Schmidt M.. (2011). Microcircuits of functionally identified neurons in the rat medial entorhinal cortex. Neuron 70, 773–786. 10.1016/j.neuron.2011.04.003 21609831

[B5] Canto C. B. Koganezawa N. Beed P. Moser E. I. Witter M. P. (2012). All layers of medial entorhinal cortex receive presubicular and parasubicular inputs. J. Neurosci. 32, 17620–17631. 10.1523/JNEUROSCI.3526-12.2012 23223285 PMC6621674

[B6] Chrobak J. J. Buzsáki G. (1994). Selective activation of deep layer (V-VI) retrohippocampal cortical neurons during hippocampal sharp waves in the behaving rat. J. Neurosci. 14, 6160–6170. 7931570 10.1523/JNEUROSCI.14-10-06160.1994 PMC6576977

[B7] Clapham D. E. (2007). SnapShot: mammalian TRP channels. Cell 129, 220.e1–220.e2. 10.1016/j.cell.2007.03.034 17418797

[B8] Congar P. Leinekugel X. Ben-Ari Y. Crépel V. (1997). A long-lasting calcium-activated nonselective cationic current is generated by synaptic stimulation or exogenous activation of group I metabotropic glutamate receptors in CA1 pyramidal neurons. J. Neurosci. 17, 5366–5379. 9204921 10.1523/JNEUROSCI.17-14-05366.1997 PMC6793810

[B9] Crowder E. A. Saha M. S. Pace R. W. Zhang H. Prestwich G. D. Del Negro C. A. (2007). Phosphatidylinositol 4,5-bisphosphate regulates inspiratory burst activity in the neonatal mouse preBötzinger complex. J. Physiol. 582, 1047–1058. 10.1113/jphysiol.2007.134577 17599963 PMC2075248

[B10] de Curtis M. Paré D. (2004). The rhinal cortices: a wall of inhibition between the neocortex and the hippocampus. Prog. Neurobiol. 74, 101–110. 10.1016/j.pneurobio.2004.08.005 15518955

[B11] Dhillon A. Jones R. S. G. (2000). Laminar differences in recurrent excitatory transmission in the rat entorhinal cortex *in vitro*. Neuroscience 99, 413–422. 10.1016/s0306-4522(00)00225-6 11029534

[B12] Egorov A. V. Hamam B. N. Fransén E. Hasselmo M. E. Alonso A. A. (2002). Graded persistent activity in entorhinal cortex neurons. Nature 420, 173–178. 10.1038/nature01171 12432392

[B13] Flower R. J. (1974). Drugs which inhibit prostaglandin biosynthesis. Pharmacol. Rev. 26, 33–67. 4208101

[B14] Fowler M. A. Sidiropoulou K. Ozkan E. D. Phillips C. W. Cooper D. C. (2007). Corticolimbic expression of TRPC4 and TRPC5 channels in the rodent brain. PLoS One 2:e573. 10.1371/journal.pone.0000573 17593972 PMC1892805

[B15] Fox A. P. Nowycky M. C. Tsien R. W. (1987). Kinetic and pharmacological properties distinguishing three types of calcium currents in chick sensory neurones. J. Physiol. 394, 149–172. 10.1113/jphysiol.1987.sp016864 2451016 PMC1191955

[B16] Gasparini S. (2011). Distance- and activity-dependent modulation of spike back-propagation in layer V pyramidal neurons of the medial entorhinal cortex. J. Neurophysiol. 105, 1372–1379. 10.1152/jn.00014.2010 21209358 PMC3074413

[B17] Gasparini S. Magee J. C. (2006). State-dependent dendritic computation in hippocampal CA1 pyramidal neurons. J. Neurosci. 26, 2088–2100. 10.1523/JNEUROSCI.4428-05.2006 16481442 PMC6674927

[B18] Gee C. E. Benquet P. Gerber U. (2003). Group I metabotropic glutamate receptors activate a calcium-sensitive transient receptor potential-like conductance in rat hippocampus. J. Physiol. 546, 655–664. 10.1113/jphysiol.2002.032961 12562994 PMC2342598

[B19] Grand T. Demion M. Norez C. Mettey Y. Launay P. Becq F.. (2008). 9-Phenanthrol inhibits human TRPM4 but not TRPM5 cationic channels. Br. J. Pharmacol. 153, 1697–1705. 10.1038/bjp.2008.38 18297105 PMC2438271

[B20] Gross S. A. Guzmán G. A. Wissenbach U. Philipp S. E. Zhu M. X. Bruns D.. (2009). TRPC5 is a Ca^2+^-activated channel functionally coupled to Ca^2+^-selective ion channels. J. Biol. Chem. 284, 34423–34432. 10.1074/jbc.M109.018192 19815560 PMC2797210

[B21] Guinamard R. Simard C. Del Negro C. (2013). Flufenamic acid as an ion channel modulator. Pharmacol. Ther. 138, 272–284. 10.1016/j.pharmthera.2013.01.012 23356979 PMC4116821

[B22] Gutnick M. J. Connors B. W. Prince D. A. (1982). Mechanisms of neocortical epileptogenesis *in vitro*. J. Neurophysiol. 48, 1321–1335. 7153795 10.1152/jn.1982.48.6.1321

[B24] Hamam B. N. Kennedy T. E. Alonso A. Amaral D. G. (2000). Morphological and electrophysiological characteristics of layer V neurons of the rat medial entorhinal cortex. J. Comp. Neurol. 418, 457–472. 10.1002/(SICI)1096-9861(20000320)418:4<457::aid-CNE7>3.0.CO;2-L 10713573

[B25] Jefferys J. G. (1998). Mechanisms and experimental models of seizure generation. Curr. Opin. Neurol. 11, 123–127. 10.1097/00019052-199804000-00008 9551292

[B26] Jones R. S. (1987). Complex synaptic responses of entorhinal cortical cells in the rat to subicular stimulation *in vitro*: demonstration of an NMDA receptor-mediated component. Neurosci. Lett. 81, 209–214. 10.1016/0304-3940(87)90000-0 2892156

[B27] Jones R. S. Heinemann U. (1988). Synaptic and intrinsic responses of medial entorhinal cortical cells in normal and magnesium-free medium *in vitro*. J. Neurophysiol. 59, 1476–1496. 2898511 10.1152/jn.1988.59.5.1476

[B28] Jones R. S. Lambert J. D. (1990a). The role of excitatory amino acid receptors in the propagation of epileptiform discharges from the entorhinal cortex to the dentate gyrus *in vitro*. Exp. Brain Res. 80, 310–322. 10.1007/bf00228158 1972681

[B29] Jones R. S. Lambert J. D. (1990b). Synchronous discharges in the rat entorhinal cortex *in vitro*: site of initiation and the role of excitatory amino acid receptors. Neuroscience 34, 657–670. 10.1016/0306-4522(90)90172-z 2162019

[B31] Koganezawa N. Gisetstad R. Husby E. Doan T. P. Witter M. P. (2015). Excitatory postrhinal projections to principal cells in the medial entorhinal cortex. J. Neurosci. 35, 15860–15874. 10.1523/JNEUROSCI.0653-15.2015 26631468 PMC6605449

[B32] Larkum M. E. Zhu J. J. Sakmann B. (2001). Dendritic mechanisms underlying the coupling of the dendritic with the axonal action potential initiation zone of adult rat layer 5 pyramidal neurons. J. Physiol. 533, 447–466. 10.1111/j.1469-7793.2001.0447a.x 11389204 PMC2278642

[B33] Launay P. Fleig A. Perraud A.-L. Scharenberg A. M. Penner R. Kinet J.-P. (2002). TRPM4 is a Ca^2+^-activated nonselective cation channel mediating cell membrane depolarization. Cell 109, 397–407. 10.1016/s0092-8674(02)00719-5 12015988

[B34] Magee J. C. Carruth M. (1999). Dendritic voltage-gated ion channels regulate the action potential firing mode of hippocampal CA1 pyramidal neurons. J. Neurophysiol. 82, 1895–1901. 10515978 10.1152/jn.1999.82.4.1895

[B35] Mantegazza M. Rusconi R. Scalmani P. Avanzini G. Franceschetti S. (2010). Epileptogenic ion channel mutations: from bedside to bench and, hopefully, back again. Epilepsy Res. 92, 1–29. 10.1016/j.eplepsyres.2010.08.003 20828990

[B36] Medinilla V. Johnson O. Gasparini S. (2013). Features of proximal and distal excitatory synaptic inputs to layer V neurons of the rat medial entorhinal cortex. J. Physiol. 591, 169–183. 10.1113/jphysiol.2012.237172 23006478 PMC3630779

[B37] Metz A. E. Jarsky T. Martina M. Spruston N. (2005). R-type calcium channels contribute to afterdepolarization and bursting in hippocampal CA1 pyramidal neurons. J. Neurosci. 25, 5763–5773. 10.1523/JNEUROSCI.0624-05.2005 15958743 PMC6724888

[B38] Mrejeru A. Wei A. Ramirez J. M. (2011). Calcium-activated non-selective cation currents are involved in generation of tonic and bursting activity in dopamine neurons of the substantia nigra pars compacta. J. Physiol. 589, 2497–2514. 10.1113/jphysiol.2011.206631 21486760 PMC3115821

[B39] Nilius B. Prenen J. Droogmans G. Voets T. Vennekens R. Freichel M.. (2003). Voltage dependence of the Ca^2+^-activated cation channel TRPM4. J. Biol. Chem. 278, 30813–30820. 10.1074/jbc.M305127200 12799367

[B40] Pace R. W. Mackay D. D. Feldman J. L. Del Negro C. A. (2007). Inspiratory bursts in the preBötzinger complex depend on a calcium-activated non-specific cation current linked to glutamate receptors in neonatal mice. J. Physiol. 582, 113–125. 10.1113/jphysiol.2007.133660 17446214 PMC2075310

[B41] Partridge L. D. Valenzuela C. F. (2000). Block of hippocampal CAN channels by flufenamate. Brain Res. 867, 143–148. 10.1016/s0006-8993(00)02275-7 10837807

[B42] Prisco G. V. D. Pearlstein E. Le Ray D. Robitaille R. Dubuc R. (2000). A cellular mechanism for the transformation of a sensory input into a motor command. J. Neurosci. 20, 8169–8176. 11050140 10.1523/JNEUROSCI.20-21-08169.2000 PMC6772722

[B43] Rall W. (1967). Distinguishing theoretical synaptic potentials computed for different soma-dendritic distributions of synaptic input. J. Neurophysiol. 30, 1138–1168. 6055351 10.1152/jn.1967.30.5.1138

[B44] Ramsey I. S. Delling M. Clapham D. E. (2006). An introduction to trp channels. Annu. Rev. Physiol. 68, 619–647. 10.1146/annurev.physiol.68.040204.100431 16460286

[B45] Scharfman H. (2002). “The parahippocampal region in temporal lobe epilepsy,” in The Parahippocampal Region: Organization and Role in Cognitive Function, eds Witter M. P. Wouterlood F. G. (Oxford: Oxford University Press), 319–338.

[B46] Schiller Y. (2004). Activation of a calcium-activated cation current during epileptiform discharges and its possible role in sustaining seizure-like events in neocortical slices. J. Neurophysiol. 92, 862–872. 10.1152/jn.00972.2003 15277598

[B47] Sørensen K. E. Shipley M. T. (1979). Projections from the subiculum to the deep layers of the ipsilateral presubicular and entorhinal cortices in the guinea pig. J. Comp. Neurol. 188, 313–333. 10.1002/cne.901880208 500861

[B48] Spruston N. McBain C. J. (2006). “Structural and functional properties of hippocampal Neurons,” in The Hippocampus Book, eds Andersen P. Morris R. Amaral D. G. Bliss T. O’Keefe J. (Oxford, New York, NY: Oxford University Press), 133–201.

[B49] Stanton P. K. Jones R. S. G. Mody I. Heinemann U. (1987). Epileptiform activity induced by lowering extracellular [Mg^2+^] in combined hippocampal-entorhinal cortex slices: modulation by receptors for norepinephrine and N-methyl-d-aspartate. Epilepsy Res. 1, 53–62. 10.1016/0920-1211(87)90051-9 2904361

[B50] Stroh O. Freichel M. Kretz O. Birnbaumer L. Hartmann J. Egger V. (2012). NMDA receptor-dependent synaptic activation of TRPC channels in olfactory bulb granule cells. J. Neurosci. 32, 5737–5746. 10.1523/JNEUROSCI.3753-11.2012 22539836 PMC3349057

[B51] Suzuki M. Morita T. Iwamoto T. (2006). Diversity of Cl^−^ channels. Cell. Mol. Life Sci. 63, 12–24. 10.1007/s00018-005-5336-4 16314923 PMC2792346

[B52] Swanson L. W. Cowan W. M. (1977). An autoradiographic study of the organization of the efferent connections of the hippocampal formation in the rat. J. Comp. Neurol. 172, 49–84. 10.1002/cne.901720104 65364

[B53] Tian J. Thakur D. P. Lu Y. Zhu Y. Freichel M. Flockerzi V.. (2013). Dual depolarization responses generated within the same lateral septal neurons by TRPC4-containing channels. Pflüg. Arch. 466, 1301–1316. 10.1007/s00424-013-1362-5 24121765 PMC3984930

[B54] Tozzi A. Bengtson C. P. Longone P. Carignani C. Fusco F. R. Bernardi G.. (2003). Involvement of transient receptor potential-like channels in responses to mGluR-I activation in midbrain dopamine neurons. Eur. J. Neurosci. 18, 2133–2145. 10.1046/j.1460-9568.2003.02936.x 14622174

[B55] Traub R. D. Wong R. K. (1982). Cellular mechanism of neuronal synchronization in epilepsy. Science 216, 745–747. 10.1126/science.7079735 7079735

[B56] Ullrich N. D. Voets T. Prenen J. Vennekens R. Talavera K. Droogmans G.. (2005). Comparison of functional properties of the Ca^2+^-activated cation channels TRPM4 and TRPM5 from mice. Cell Calcium 37, 267–278. 10.1016/j.ceca.2004.11.001 15670874

[B57] Vazquez G. Wedel B. J. Aziz O. Trebak M. Putney J. W. Jr. (2004). The mammalian TRPC cation channels. Biochim. Biophys. Acta 1742, 21–36. 10.1016/j.bbamcr.2004.08.015 15590053

[B23] von Bohlen Und Halbach O. Hinz U. Unsicker K. Egorov A. V. (2005). Distribution of TRPC1 and TRPC5 in medial temporal lobe structures of mice. Cell Tissue Res. 322, 201–206. 10.1007/s00441-005-0004-4 16044320

[B58] Walther H. Lambert J. D. Jones R. S. Heinemann U. Hamon B. (1986). Epileptiform activity in combined slices of the hippocampus, subiculum, and entorhinal cortex during perfusion with low magnesium medium. Neurosci. Lett. 69, 156–161. 10.1016/0304-3940(86)90595-1 3763042

[B59] Wilson C. L. Khan S. U. Engel J. Jr. Isokawa M. Babb T. L. Behnke E. J. (1998). Paired pulse suppression and facilitation in human epileptogenic hippocampal formation. Epilepsy Res. 31, 211–230. 10.1016/s0920-1211(98)00063-1 9722031

[B60] Wong R. K. Traub R. D. Miles R. (1986). Cellular basis of neuronal synchrony in epilepsy. Adv. Neurol. 44, 583–592. 3706021

[B61] Yau H.-J. Baranauskas G. Martina M. (2010). Flufenamic acid decreases neuronal excitability through modulation of voltage-gated sodium channel gating. J. Physiol. 588, 3869–3882. 10.1113/jphysiol.2010.193037 20724367 PMC3000579

[B62] Zhang Z. Reboreda A. Alonso A. Barker P. A. Séguéla P. (2011). TRPC channels underlie cholinergic plateau potentials and persistent activity in entorhinal cortex. Hippocampus 21, 386–397. 10.1002/hipo.20755 20082292

